# Data documenting the potential distribution of *Aedes aegypti* in the center of Veracruz, Mexico

**DOI:** 10.1016/j.dib.2016.12.014

**Published:** 2016-12-16

**Authors:** Israel Estrada-Contreras, César A. Sandoval-Ruiz, Fredy S. Mendoza-Palmero, Sergio Ibáñez-Bernal, Miguel Equihua, Griselda Benítez

**Affiliations:** a Red de Ambiente y Sustentabilidad, Instituto de Ecología, A.C., Carretera Antigua a Coatepec No. 351 El Haya, Xalapa, C.P. 91070 Veracruz, México; b Laboratorio de Artropodología y Salud, Escuela de Biología, Benemérita Universidad Autónoma de Puebla. Puebla, México. Blvd. Valsequillo y Av. San Claudio Edificio 112-A, Ciudad Universitaria Col. Jardines de San Manuel, C.P. 72570 Puebla, Mexico; c Departamento de Vigilancia Epidemiológica, Subdirección de Epidemiología, Servicios de Salud de Veracruz (SESVER), Ernesto Ortiz Medina No. 3 Col. Obrero Campesina, Xalapa, C.P. 91120 Veracruz, México

**Keywords:** Ecological niche modelling, Vector, MaxLike, Mobility-oriented parity

## Abstract

The data presented in this article are related to the research article entitled “Establishment of *Aedes aegypti* (L.) in mountainous regions in Mexico: Increasing number of population at risk of mosquito-borne disease and future climate conditions” (M. Equihua, S. Ibáñez-Bernal, G. Benítez, I. Estrada-Contreras, C.A. Sandoval-Ruiz, F.S. Mendoza-Palmero, 2016) [1]. This article provides presence records in shapefile format used to generate maps of potential distribution of *Aedes aegypti* with different climate change scenarios as well as each of the maps obtained in raster format. In addition, tables with values of potential distribution of the vector as well as the average values of probability of presence including data of the mosquito incidence along the altitudinal range.

**Specifications Table**

**Table:** 

Subject area	Biology and climate change
More specific subject area	Ecological niche modelling
Type of data	Maps, tables and figures
How data was acquired	A dataset sampling for the state of Veracruz: 100 records of *Aedes aegypti* from previous surveys, 167 also records provided by the Health Authority for Region V, state of Veracruz and seven records from our sampling data. Potential distribution maps of *Aedes aegypti* were obtained using the packages “maxlike” ver. 0.1–5, “raster” ver. 2.3–12, “rgdal” ver. 0.9–1, “sp” ver. 1.0–16 and “tcltk2” ver. 1.2–10, in the software R ver. 3.1.2. In addition, a geographic information system was used to analyze the maps obtained.
Data format	Shapefile (.shp) and Excel (.xlsx)
Data source location	Veracruz, Mexico
Data accessibility	Data are available in this article

**Value of the data**

• Presence records over a gradient including current boundary conditions is interesting to assess current *Aedes aegypti* distribution expansion.
• Potential distribution mosquito coverage is useful in planning future strategies to face the human risks produced by*Aedes aegypti* expansion.
• The potential distribution of*Aedes aegypti*could be used to compare the output of other algorithms used in ecological niche modeling.

## 1 Data

**Map. 1:**
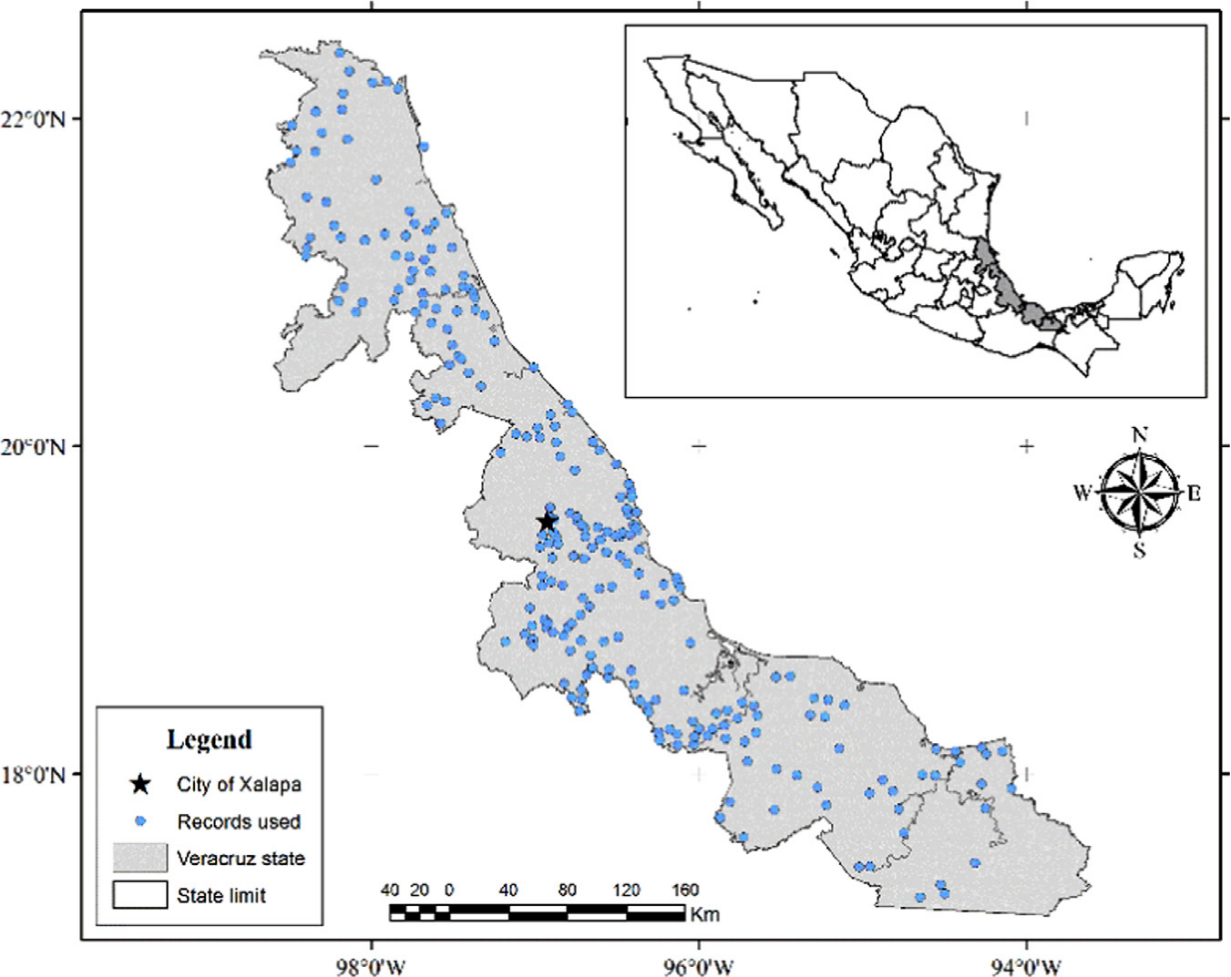
Occurrence records of *Aedes aegypti*.

The dataset of this article provides information about occurrence records used to generate the potential distribution maps of *Aedes aegypti*, we produced a series of maps about this. This maps are presented and discussed in Equihua et al. [1]. The map included (Map 1) is the spatial distribution of the records used to generate *Aedes aegypti* potential distribution models (shared in shapefile format). The following five maps are the potential distribution obtained under the different scenarios of climate change we explored. Map 2 is the current potential distribution, Map 3 is the RCP 4.5 to 2030 scenario, Map 4 is the RCP 8.5 to 2030 scenario, Map 5 is the RCP 4. 5 to 2080 scenario and Map 6 is the RCP 8.5 to 2080 scenario. They are shared in raster geo-TIF format. Table 1, Table 2, Table 3 show information about the area, probability of occurrence and potential altitudinal presence in different altitudinal ranges where the potential presence of mosquito is projected (they are shared in.xlsx format).

## 2 Experimental design, materials and methods

We developed ecological niche models of *Ae. aegypti* for the state of Veracruz with a total of 274 verified records. Seven records from our sampling data, 100 records from previous surveys and 167 records provided by the Health Authority for Region V, state of Veracruz. We verified all of them for geographic accuracy with on-screen visual inspection using a Geographic Information System image.

To develop potential distribution models of *Ae. aegypti* we used bioclimate variables for current conditions [2] and projected to future [3]. The bioclimate variables used were Bio5: maximum temperature of the warmest month, Bio6: minimum temperature of the coldest month, Bio13: precipitation of the wettest month and Bio14: precipitation of the driest month.

The results of correlation analysis for 19 bioclimate variables indicate that the four variables selected highly correlate with 2 principal component that account for almost 92% of the variability in the data. For each projection into future conditions, we used two Representative Concentration Pathways (RCP): RCP 4.5 and RCP 8.5, which refer to the possible range of radiative forcing values in the year 2100 relative to pre-industrial values, expressed in W/m2 [4].

We standardized all bioclimate variables (current and future) with their corresponding current layer, i. e. for the projected value of each variable we subtracted the mean and then divided it by the standard deviation of the current data subset. We used the MaxLike software package [5] to generate potential distribution maps and we used the packages “maxlike” ver. 0.1–5, “raster” ver. 2.3–12, “rgdal” ver. 0.9–1, “sp” ver. 1.0–16 and “tcltk2” ver. 1.2–10, in the software R ver. 3.1.2.

Then, we randomly selected 65% of the records for training and the remaining 35% for cross-validation each of the 1000 times the process was repeated with the current conditions dataset. The resulting models were deemed adequate, according to Estrada-Contreras et al. [6], if they satisfied the following criteria: a) convergence occurred, b) they had no missing data, and c) proportion of errors of omission was less than or equal to 10. The model coefficients were then used to project the species’ future niche. The resulting models were ranked by how well they matched the relative occurrence area (ROA) [7] values. We chose 10 models around the statistical median that had an average probability of presence obtained with validation records closest to 1, since theoretically the average of this value should be 1. Then we produced a consensus map averaging these 10 maps (the same models set for current and future conditions).

The minimum value of probability of presence was considered indicative of the likely presence of *Ae. aegypti*, and was obtained by extracting values from the potential distribution map to current conditions with the coordinates of all the records used to generate the models (training and validation). To further evaluate the current presence model we used partial ROC [8] by randomly selecting 35% of the records used to generate the models.

Although ecological niche models were generated for surface analysis of the entire state of Veracruz, elevation increase and changes in the probability of occurrence were conducted only in the rectangle that has its diagonal vertices at points 97 °35’55.78’’W and 20 °28’20.67’’N, and 95 °49’31.07’’W and 18 °39’41.6’’N, which covers an area of 28,167.58 km^2^. To identify whether the analysis area has combinations of environmental variables similar to those of today, the "Mobility-Oriented Parity"(MOP) tool [9] was used.

## Conflict of Interest

There is no conflict of interest.

## Figures and Tables

**Table 1:** Potential distribution area of *Aedes aegypti* in central Veracruz, Mexico.

**Altitudinal range (m)**	**Area (km**^**2**^**)**				
	**Current**	**Near future 4.5**	**Near future 8.5**	**Distant future 4.5**	**Distant future 8.5**
0–100	5963.24	2816.5	2900.78	901.28	NA
101–200	2500.02	1547.14	1598.74	389.58	5.16
201–300	1406.96	1186.8	1232.38	382.7	43
301–400	1079.3	1096.5	1099.08	479.02	135.88
401–500	902.14	911.6	911.6	829.04	187.48
501–600	748.2	749.06	749.06	745.62	180.6
601–700	634.68	634.68	634.68	633.82	174.58
701–800	552.12	552.12	552.12	552.12	168.56
801–900	578.78	578.78	578.78	578.78	393.88
901–1000	521.16	521.16	521.16	521.16	449.78
1001–1100	480.74	489.34	489.34	489.34	444.62
1101–1200	467.84	495.36	495.36	495.36	468.7
1201–1300	418.82	454.94	455.8	455.8	430.86
1301–1400	374.96	429.14	435.16	437.74	405.92
1401–1500	261.44	344.86	348.3	360.34	337.98
1501–1600	116.96	257.14	264.02	302.72	281.22
1601–1700	54.18	227.9	235.64	276.06	260.58
1701–1800	8.6	155.66	172.86	229.62	230.48
1801–1900	0.86	84.28	102.34	210.7	245.96
1901–2000	NA	12.9	24.94	157.38	208.98
2001–2100	NA	0.86	1.72	61.92	155.66
2101–2200	NA	0.86	1.72	18.06	112.66
2201–2300	NA	NA	NA	0.86	56.76
2301–2400	NA	NA	NA	0.86	25.8
2401–2500	NA	NA	NA	NA	5.16
2501–2600	NA	NA	NA	NA	1.72

**Table 2:** Mean probability of occurrence of *Aedes aegypti* in central Veracruz, Mexico.

**Altitudinal range (m)**	**Mean probability of occurrence**				
	**Current**	**Near future 4.5**	**Near future 8.5**	**Distant future 4.5**	**Distant future 8.5**
0–100	0.94	0.85	0.85	0.81	NA
101–200	0.94	0.79	0.80	0.79	0.29
201–300	0.99	0.71	0.73	0.85	0.44
301–400	0.99	0.86	0.89	0.76	0.58
401–500	1.00	0.97	0.98	0.73	0.69
501–600	0.99	1.00	1.00	0.87	0.67
601–700	1.00	1.00	1.00	0.96	0.73
701–800	1.00	1.00	1.00	0.96	0.82
801–900	0.99	1.00	1.00	0.98	0.71
901–1000	0.99	1.00	1.00	0.99	0.85
1001–1100	0.98	1.00	1.00	1.00	0.87
1101–1200	0.98	1.00	1.00	1.00	0.87
1201–1300	0.98	0.98	0.99	0.99	0.86
1301–1400	0.95	0.94	0.95	0.96	0.90
1401–1500	0.84	0.93	0.95	0.96	0.93
1501–1600	0.86	0.90	0.90	0.89	0.92
1601–1700	0.73	0.87	0.89	0.88	0.94
1701–1800	0.61	0.77	0.79	0.87	0.93
1801–1900	0.31	0.67	0.69	0.85	0.88
1901–2000	NA	0.45	0.46	0.76	0.89
2001–2100	NA	0.71	0.68	0.65	0.78
2101–2200	NA	0.54	0.51	0.58	0.58
2201–2300	NA	NA	NA	0.50	0.45
2301–2400	NA	NA	NA	0.46	0.34
2401–2500	NA	NA	NA	NA	0.29
2501–2600	NA	NA	NA	NA	0.28

**Table 3:** Mean altitude of potential presence of *Aedes aegypti* in central Veracruz, Mexico.

**Altitudinal range (m)**	**Mean altitude of potential presence (m)**				
	**Current**	**Near future 4.5**	**Near future 8.5**	**Distant future 4.5**	**Distant future 8.5**
0–100	36.94	46.00	45.34	31.47	NA
101–200	143.53	141.71	142.60	150.82	161.00
201–300	248.39	252.13	251.84	250.04	259.64
301–400	348.76	348.83	348.77	354.33	354.31
401–500	449.68	449.63	449.63	451.98	451.68
501–600	549.55	549.51	549.51	549.56	551.56
601–700	648.64	648.64	648.64	648.69	649.42
701–800	749.71	749.71	749.71	749.71	752.05
801–900	850.45	850.45	850.45	850.45	854.55
901–1000	949.34	949.34	949.34	949.34	951.34
1001–1100	1050.74	1050.82	1050.82	1050.82	1051.13
1101–1200	1152.24	1152.89	1152.89	1152.89	1153.48
1201–1300	1248.68	1249.10	1249.11	1249.11	1248.54
1301–1400	1349.18	1349.42	1349.64	1349.77	1350.19
1401–1500	1445.48	1447.89	1447.86	1448.25	1449.00
1501–1600	1548.25	1550.11	1550.19	1551.14	1550.78
1601–1700	1636.68	1648.68	1648.72	1649.72	1650.83
1701–1800	1726.40	1746.26	1747.41	1749.27	1750.32
1801–1900	1824.00	1839.97	1841.95	1848.89	1848.62
1901–2000	NA	1931.73	1934.97	1947.19	1952.33
2001–2100	NA	2001.00	2030.00	2039.53	2051.32
2101–2200	NA	2182.00	2146.50	2133.19	2146.58
2201–2300	NA	NA	NA	2212.00	2235.58
2301–2400	NA	NA	NA	2365.00	2346.27
2401–2500	NA	NA	NA	NA	2429.17
2501–2600	NA	NA	NA	NA	2515.50
