# Supplementary figures and images for: Data documenting the potential distribution of Aedes aegypti in the center of Veracruz, Mexico
Source: Data Brief. 2016 Dec 16;10:432–7. doi: 10.1016/j.dib.2016.12.014 (PMC5198849; doi:10.1016/j.dib.2016.12.014)

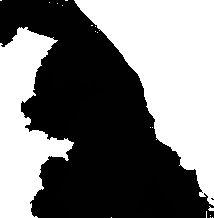

Supplement: Supplementary file 1 — Supplementary material [file mmc1.zip › supplementary file/supplementary map 2.tif]

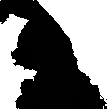

Supplement: Supplementary file 1 — Supplementary material [file mmc1.zip › supplementary file/supplementary map 2.tif.ovr]

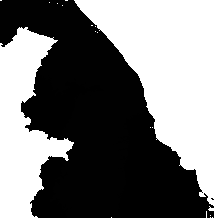

Supplement: Supplementary file 1 — Supplementary material [file mmc1.zip › supplementary file/supplementary map 3.tif]

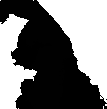

Supplement: Supplementary file 1 — Supplementary material [file mmc1.zip › supplementary file/supplementary map 3.tif.ovr]

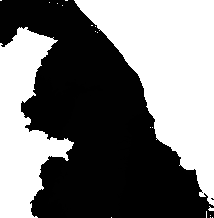

Supplement: Supplementary file 1 — Supplementary material [file mmc1.zip › supplementary file/supplementary map 4.tif]

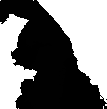

Supplement: Supplementary file 1 — Supplementary material [file mmc1.zip › supplementary file/supplementary map 4.tif.ovr]

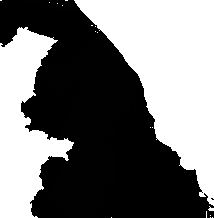

Supplement: Supplementary file 1 — Supplementary material [file mmc1.zip › supplementary file/supplementary map 5.tif]

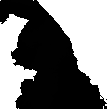

Supplement: Supplementary file 1 — Supplementary material [file mmc1.zip › supplementary file/supplementary map 5.tif.ovr]

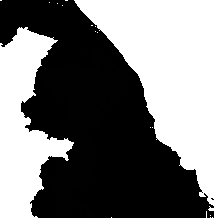

Supplement: Supplementary file 1 — Supplementary material [file mmc1.zip › supplementary file/supplementary map 6.tif]

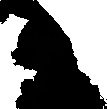

Supplement: Supplementary file 1 — Supplementary material [file mmc1.zip › supplementary file/supplementary map 6.tif.ovr]
